# Single-port laparoscopic appendectomy using a needle-type grasping forceps for acute uncomplicated appendicitis in children: Case series

**DOI:** 10.1016/j.ijscr.2020.03.040

**Published:** 2020-05-06

**Authors:** Yang Chen, Jie-Qing Yuan, Shi-Gang Guo, Zhen-Jiang Yang

**Affiliations:** Department of General Surgery, Chaoyang Central Hospital, Chaoyang, China

**Keywords:** Single-port, Laparoscopy, Appendectomy, Children, Uncomplicated appendicitis, Case series

## Abstract

**Introduction:**

Single-port laparoscopy has been used in any areas of surgery, including appendectomy, to reduce the operative stress and enhance postoperative recovery procedure. This paper introduces our attempt to perform single-port laparoscopic appendectomy using conventional laparoscoopic instruments and a needle-type grasping forceps (SLAN), which has dominant advantage in cosmetic appearance.

**Methods:**

We report six pediatric patients who underwent SLAN for uncomplicated appendicitis from April to November 2019. SLAN was performed transumbilically, while conventional laparoscopic instruments and needle-type grasping forceps were both used. After routine intracorporeal appendectomy was completed, the pathological appendix was extracted through the single-port site, while a 10 mm trocar was used to avoid incision infectious. Clinical data and postoperative follow-up data were collected and analyzed to evaluate the feasibility, safety, and clinical outcomes of this novel technique.

**Results:**

SLAN was successfully performed in all six pediatric patients. The median operative time, first exhaust time after surgery, pain score of postoperative day 1, and postoperative hospital stay were 69 (range, 50−85) min, 1.33 (range, 1–2) d, 0.83 (range, 0–3) score, 1.5 (range, 1–2) d, respectively. Neither intraoperative nor postoperative complications were noted, while no incision infectious, adhesive intestinal obstruction, and abdominal abscess were observed with 2–9 months follow up.

**Discussion:**

Though there are many methods to perform single-port laparoscopic appendectomy, the use of needle grasping forceps in laparoscopic appendectomy has been confirmed a new choice for uncomplicated appendicitis in children.

**Conclusion:**

SLAN is a feasible and safe technique to treat acute uncomplicated appendicitis in children. To be emphasized, surgeons must strictly grasp the indications for this surgery.

## Highlights

• Acute appendicitis is the most common between the ages of 10 and 20 years.
• Our centre performed a new technology of single-port laparoscopic appendectomy using a needle-type grasping forceps (SLAN).
• SLAN has advantages of favourable cosmetic results, minimal trauma, and enhanced postoperative recovery.

## 1 Introduction

Acute appendicitis is the most common abdominal surgical emergency in the world, who can affect people of any age but is most common between the ages of 10 and 20 years [1]. McBurney firstly reported open appendectomy in 1891 [2], which has been the main operation choice for a time. Recently, more and more concerns have been concentrated to the application of laparoscopy, especially single-port laparoscopy, in the treatment of acute appendicitis, relating to the rising requirement of mini-trauma and cosmetic appearance. Our centre successfully performed single-port laparoscopic appendectomy using a needle-type grasping forceps (SLAN) for a pediatric patient with acute simplex appendicitis in April 2019. SLAN was performed with a 1 cm transumbilical incision, while conventional laparoscopic instruments and a needle-type grasping forceps were both used. The main operative procedure is similar to conventional three-port laparoscopic appendectomy, which can shorten the learning curve, and warrant safety, along with the advantages of favourable cosmetic results, minimal trauma, and enhanced postoperative recovery. In this report, we introduced our experience with SLAN for the treatment of six pediatric patients with uncomplicated appendicitis from April to November 2019, and assessed the feasibility and safety of this technique.

## 2 Materials and methods

Between April to November 2019, six pediatric patients with uncomplicated appendicitis (including acute simplex and purulent appendicitis) underwent emergent SLAN in our centre. All patients were provided informed consent prior to undergoing surgery. All patients were diagnosed with acute appendicitis based on their medical history, physical sign, chemical examination results, abdominal ultrasound and computer-tomography. Patients with preoperative evidence of gangrene or perforated appendicitis, severe ascites or adhesion were excluded. The median age and BMI were 10.7 (range, 6–14) y, 18.40 (range, 14.57–21.48) kg/m2. The operation was performed by the same one attending surgeon in our center, who has adequate experiences on laparoscopic procedure. The research work has been reported in line with the PROCESS criteria [3].

**Fig. 1:**
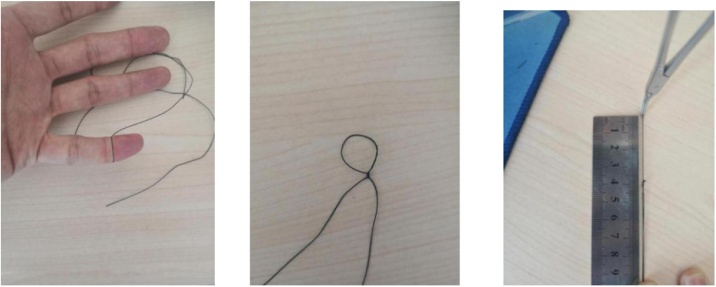
The umbilical port size who just permits a little finger passing through may be suitable.

**Fig. 2:**
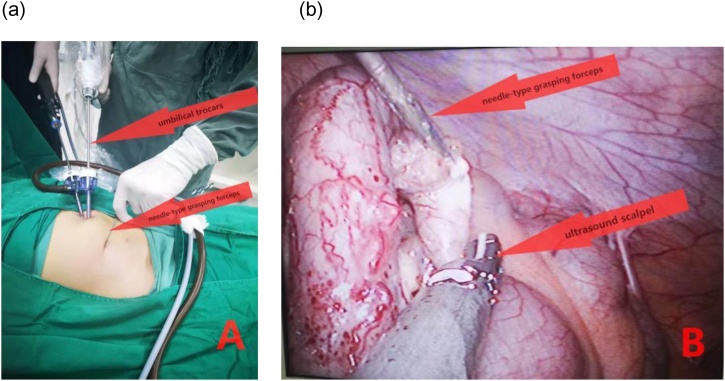
(a) Placement of the umbilical trocars and needle-type grasping forceps. (b) Laparoscopic view showing the procedure of dissociating the mesentery of appendix with assist of needle-type grasping forceps.

Every patient was required to ensure fasting at least 6 h and evacuate bladder to ensure the bladder flatulent, while the insertion of gastric canal and urinary catheter is unnecessary. Under general endotracheal anesthesia, the patient was positioned in supine position, and laparoscopic video screen was placed on the right side, master surgeon standed on bottom-left, assistant surgeon standed on top-left. When all preparations were ready, one umbilical port (about 10 mm) was dissected by open pathway. Actually, the port size who just permits a little finger passing through may be suitable, because CO2 air leakage may occur if the port is dissected longer (Fig. 1). Two 5 mm disposable trocars were inserted into abdominal cavity through the umbilical port to be used of observation and procedure (Fig. 2), while two seventh surgical sutures were used to seal the port and fix trocars. The intraoperative diagnosis of acute uncomplicated appendicitis (including acute simplex and purulent appendicitis) was confirmed by laparoscopic exploration. Then the patient was tilted 300 to leg, and titled 300 to left side. A 50 ml injector pinhead was sticked into abdominal wall on the McBurney site, which can help needle-type grasping forceps be inserted into abdominal cavity easily. By the assistance of the needle-type grasping forceps to drag appendix lumen, the mesentery of appendix was dissociated until the root of appendix revealed. Two seventh surgical sutures were used to ligate and slender the root of appendix, and a green Hem-o-lock clip was used to seal the root appendix. Another seventh surgical sutures was used to ligate distal appendix lumen, to be ready for search of appendix when CO2 air in abdomen was evacuated in the end. Appendix was cut by ultrasound scalpel with distance about 3−5 mm from the root of appendix. Then ascites were sucked until clean. When carbasus and all other surgical equipments were confirmed integrated, and no bleeding happened in neither umbilical port nor McBurney site. Then the seventh surgical suture in the distal appendix was extracted through 5 mm trocar by laparoscopic elastic separating plier, then a 10 mm disposable trocar was inserted into umbilical port following anterior two 5 mm trocars were taken out. In the end, the pathological appendix was extracted through 10 mm disposable trocar to avoid incision infectious. Umbilical incision was sutured with one 3−0 absorptive suture (Table 1).

**Table 1:** Clinical characteristic, operative and postoperative data of pediatric patients undergoing single-port laparoscopic appendectomy using a needle-type grasping forceps.

Patient	Sex	Age (yr)	BMI (kg/m2)	Diagnosis	Appendix length (cm)	Appendix diameter (cm)	ASA^②^ stage	Operative time (min)	First exhaust time after surgery (d)	VAS score of POD1	SIHD	Postoperative hospital stay (d)
1	M	6	14.54	APA	7.3	1	II	70	2	2	Grade A	2
2	M	9	17.54	APA	7.5	1	II	75	1	0	Grade A	1
3	M	10	20.76	ASA^①^	8	0.6	II	50	1	0	Grade A	1
4	F	11	14.98	ASA^①^	6	0.4	II	68	1	0	Grade A	1
5	M	14	21.08	APA	7.5	1	I	85	2	3	Grade A	2
6	M	14	21.48	ASA^①^	5.2	0.6	II	67	1	0	Grade A	2

BMI: body mass index; ASA^①^: acute simplex appendicitis; APA: acute purulent appendicitis; ASA^②^: American Society of Anesthesiologists; VAS: visual analogue scale, score range 0–3 means slight and tolerable pain [21]; POD1: postoperative day 1； SIHD: surgical incision healing grades; Grade A: surgical incision healing excellent, without any side effects.

## 3 Results

All six pediatric patients were successfully performed SLAN, and none of them was converted to open surgery. The operation time was 50−85 min; the postoperative hospitalization was 1–2 d; the first exhaust time after surgery was 1–2 d. The umbilical incision is small and covert, without obvious scars are visible. The puncture point of needle grasping forceps in the right lower abdomen is about 2 mm, which is not necessary to be sutured because of retracting properties of the skin. During the follow-up of 2–9 months, none complications were observed, such as incisional infection, adhesive intestinal obstruction, and abdominal abscess formation. Satisfied feedback was received from all the patients and their family.

## 4 Discussion

Acute appendicitis is the most common surgical abdomen in the world with a high incidence [1]. Although there is still an international controversy regarding the choice of conservative treatment or active surgical interference, surgery is still considered to be an active and effective treatment [[4], [5], [6], [7], [8]]. Since Semm firstly reported laparoscopic appendectomy in 1983 [9], and Gans and Berci introduced laparoscopic appendectomy into pediatric surgery in 1973 [10], laparoscopic appendectomy has been confirmed to be predominant, including safe, effective, minimally invasive, and recovered quickly after surgery. In recent years, with the development of laparoscopic technology, single-port laparoscopic appendectomy has received more attention in the field of pediatric surgery and has been proven to be safe and effective [[11], [12], [13]]. In general, pediatric patients are small, whose parents have higher requirements for aesthetic incision. On the premise of ensuring the safety and effectiveness of the operation, reducing the incision as much as possible and reducing surgical stress will greatly improve the satisfaction of patients and their families. Based on this, our center attempted to make a 1 cm incision along the natural folds under the umbilicus and simultaneously inserted two 5 mm trocars. The needle grasping forceps was a mini instrument which was initially used in single-port laparoscopic appendectomy in children. In order to complete the operation smoothly, our centre introduced needle grasping forceps in single-port laparoscopic appendectomy which can assist in clamping the appendix and knotting. This procedure can effectively reduce the length of the incision without affecting the surgical outcomes.

**Fig. 3:**
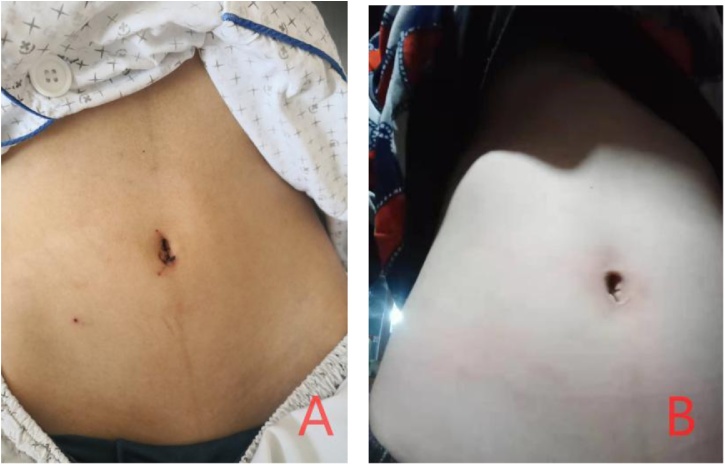
(a) Incision healing status on the first day affter surgery. (b) Incision healing status on 9 months after surgery.

Though there are many methods to perform single-port laparoscopic appendectomy, SLAN is really a progress with the following advantages: Firstly, the incision is small and covert, and postoperative appearance is excellent. The incision is selected on natural folds along the lower umbilical edge, with about 1 cm length, which plus the puncture point of the needle-type grasping forceps with a total length of about 1.2 cm. However, most of the previously reported single-port umbilicus incisions are about 1.5–2.5 cm [14,15], while the total length of traditional three-port methods is about 2.0–2.5 cm. In this comparison, SLAN showed minimal incision and surgical stress. Meanwhile, no obvious scars were observed 9 months after surgery, and the cosmetic appearance is excellent according to the follow-up result (Fig. 3). Secondly, SLAN can obviously shorten hospital stay and enhance postoperative recovery procedure. Previous studies have showed that the traditional single-port laparoscopic appendectomy requires 44.4 h hospital stay [16], in contrast, SLAN requires only 1.5 days postoperative hospital day, and recovered gas on about 1–2 days after surgery, which is in line with the concept of enhanced recovery after surgery [17]. Thirdly, the needle-type grasping forceps which was initially applied in single-port laparoscopic hernia repair, is similar to conventional laparoscopic elastic separating plier, as well as the use of conventional laparoscopic instruments, the learning curve is shortened largely. Fourthly, Hem-o-lock clip has been confirmed to be effective in laparoscopic appendectomy [18]. Thus green Hem-o-lock clip which was used in SLAN to clamp the root of the appendix with can avoid postoperative appendix stump fistula, this process is safer and more reliable than conventional suture ligation. Meanwhile, retrieval bag has been proved to reduce the risk of intra-abdominal infection during laparoscopic appendectomy [19], although retrieval bag cannot be inserted into abdominal cavity in SLAN because of the limitation of 5 mm trocar, we extracted the appendix through a 10 mm disposable trocar to avoid incision being contaminated by pathological appendix, and fortunately, no postoperative incision infections or abdominal abscesses feedback has been received in all the six patients by 2–9 months follow-up.

**Fig. 4:**
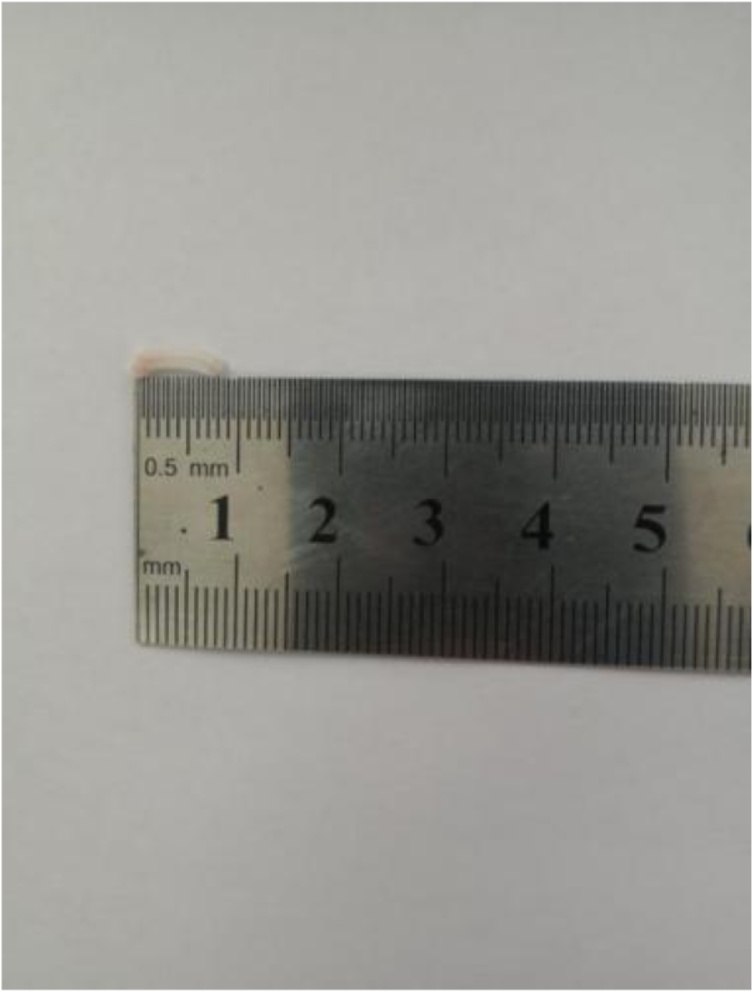
Length of green Hem-o-lock clip on clamping status.

Undoubtedly, any surgery has its limitations. To our experience, the following announcements should be emphasized: Firstly, Because the pathway of procedure and the observation shared the same umbilical port, "chopstick effect" occured inevitablely. In order to solve this trouble, the assistant need to move with the master surgeon in the same direction, to fully expose the operative field by adjusting the laparoscopic 30° bevel direction, which can make the operation comfortable and efficient. Secondly, because of lack of suitable laparoscopic instruments for SLAN, CO2 air leakage happened frequently, especially in the initial cases, which may greatly interfere surgical process. After continuous accumulation and improvement of the surgical experience, the umbilical port size who just permits a little finger passing through is favourable for avoiding "leakage" phenomenon (Fig. 2). Thirdly, because of the limitation of the tiny needle-type grasping forceps, patients with severe abdominal infection and severe abdominal adhesions were not recommended, though abdominal wall trauma and operative stress can effectively be reduced. Fourthly, the length of the green Hem-o-lock clip after closing is about 8 mm (Fig. 4), considering of the limitation of 5 mm trocar, any other longer Hem-o-lock clips cannot be inserted into abdominal cavity, so the diameter of the root of the appendix larger than 8 mm after "slimming" may induce incomplete clamping, which is not suitable for SLAN. Of course, we also considered to expand the umbilical incision to 1.5 cm, so that one 10 mm and one 5 mm trocar can be inserted into abdominal cavity respectively, if that, the clamping effect is equivalent to the three-hole method, which can expand the surgery indications. However, there is no doubt that this method will increase the length of the umbilical incision, the cosmetic effect is not good, and the postoperative stress is larger, which is contrary to our original intention. Fifthly, the abdominal drainage tube cannot be retained for the limitation of only one umbilical incision, therefore, preoperative assessment of abdominal infection and the diameter of the appendix is particularly important. Previous studies have emphasized the importance of preoperative abdominal CT examination to identify acute simple appendicitis and complex appendix [20]. Therefore, for patients without radiological contraindications, we recommends perfecting abdominal CT before surgery. If abdominal CT indicates a severe abdominal infection or adhesion, or the root of the appendix is too thick to be clamped completely by a green Hem-o-lock clip, SLAN should be decisively abandoned after the intraoperative exploration, and the conventional three-port procedure or open surgery should be performed instead, depending on the condition. In a word, proper preoperative examination and evaluation should be completed, rational cases should be selected, in order to maximize the benefits for patients.

## 5 Conclusion

SLAN is a feasible and safe technique to treat acute uncomplicated appendicitis in children. Surgeons must strictly grasp the surgery indications and select suitable patients. Randomized controlled trials with larger samples and postoperative follow-up data need to be carried out in the future.

## Conflicts of interest

None

## Sources of funding

None

## Ethical approval

No ethical approval was necessary since this paper describes a retrospective research of the procedure of usual single-port laparoscopic appendectomy with conventional instruments, and the needle-type grasping forceps has been widely used in single-port lapaorscopic high ligation of hernia sac in children, in addition, the main surgery procedure is similar to conventional three-port laparoscopic appendectomy (not considered as a ‘first-in-man’ study).

## Consent

Written informed consent was obtained from all patients for publication of this case series. A copy of all the written consents is available for review by the Editor-in-Chief of this journal on request.

## Author contribution

Y Chen - study design, data collection, data analyse, writing, and review.

JQ Yuan - study design, writing, and review;

SG Guo, ZJ Yang- data collection, and review.

## Registration of research studies

1. Name of the registry: Researchregistry

2. Unique identifying number or registration ID: researchregistry 5430

3. Hyperlink to your specific registration (must be publicly accessible and will be checked): https://www.researchregistry.com/browse-the-registry#home/

## Guarantor

Y Chen

## Provenance and peer review

Editorially reviewed, not externally peer-reviewed
